# Optimal Timing of Inguinal Hernia Repair in Premature Infants: A Retrospective Study

**DOI:** 10.3390/children13010160

**Published:** 2026-01-22

**Authors:** Joshua Z. E. Yau, Paul C. Y. Chang, Nien-Lu Wang, Jin-Cherng Sheu, Hsuan Huang, Yi-Ting Yeh

**Affiliations:** 1 Division of Pediatric Surgery, MacKay Memorial Hospital, Taipei 104217, Taiwan; joshua.3106@mmh.org.tw (J.Z.E.Y.); nlw@mmh.org.tw (N.-L.W.); jin.4324@mmh.org.tw (J.-C.S.); smilefortw.4957@mmh.org.tw (H.H.); yitingyeh.6429@mmh.org.tw (Y.-T.Y.); 2 Department of Medicine, MacKay Medical University, New Taipei City 25245, Taiwan

**Keywords:** inguinal hernia, herniorrhaphy, postoperative complications, premature infants, respiratory insufficiency

## Abstract

**Highlights:**

**What are the main findings?**

- Early inguinal hernia repair in premature infants with a PMA of <38 weeks is associated with a higher risk of postoperative respiratory complications
- Preoperative oxygen dependence and body weight < 2.8 kg at the time of surgery are the main contributing risk factors

**What are the implications of the main findings?**

- While advances in anesthesia and surgical techniques facilitate earlier intervention, the decision to proceed with repair before hospital discharge (usually around 2.5 to 2.8 kg) requires cautious risk stratification
- If early repair is chosen, postoperative protocols must be adjusted to include a prolonged observation period to ensure the timely management of potential respiratory complications

**Abstract:**

**Background/Objectives**: The optimal timing for inguinal hernia repair in premature infants remains controversial. Most premature patients in our institution undergo repair just before discharge. This study evaluates postoperative complications in premature patients and proposes the optimal timing for hernia repair. **Methods**: A retrospective single-center review was conducted between January 2020 and November 2023. All infants undergoing hernia repair as inpatients under 50 weeks postmenstrual age (PMA) were included. Data collected included demographic details, perioperative characteristics, and postoperative outcomes. **Results**: A total of 202 patients were analyzed. Forty-five patients underwent surgery before 38 weeks PMA (early group), and 157 after 38 weeks PMA (late group). The early group had significantly lower gestational age, lower body weight, and more comorbidities. Postoperative respiratory complications were more frequent in the early group. **Conclusions**: Repair after 38 weeks PMA is associated with fewer respiratory complications while earlier repair increases transient airway support requirements.

## 1. Introduction

Inguinal hernia (IH) is among the most common surgical conditions in the pediatric population. The incidence is approximately 3–5% in term infants and increases substantially to nearly 30% in premature infants [1,2]. Surgical repair is indicated to prevent bowel incarceration [3]; however, determining the optimal timing for surgery in premature infants remains challenging due to the balance between anesthetic risk, respiratory immaturity, and the risk of hernia incarceration [4,5,6,7,8].

Several studies have suggested that younger postmenstrual age (PMA) at the time of repair is associated with an increased risk of postoperative complications, particularly respiratory insufficiency [9,10]. Nevertheless, most prior research evaluated PMA ranges older than those typically encountered at our institution, where earlier repair is frequently considered. These variations underscore the importance of establishing evidence-based guidance tailored to premature infants.

The aim of this study is to assess the optimal PMA for elective inguinal hernia repair in premature infants by analyzing postoperative respiratory complications, surgical outcomes, and risk factors. This study further evaluates whether earlier repair increases the risk of adverse events and whether specific clinical indicators may guide individualized decision-making for the timing of surgery.

## 2. Materials and Methods

This retrospective single-center study reviewed premature infants diagnosed with inguinal hernia who underwent inpatient elective surgical repair between January 2020 and November 2023 at MacKay Memorial Hospital. Premature infants were defined as those born before 37 weeks of gestation. Exclusion criteria included infants with PMA > 50 weeks, those who underwent outpatient procedures, and those who had concomitant major operations. All procedures were performed by board-certified pediatric surgeons. Institutional Review Board approval was obtained (IRB No. 25MMHIS024e).

Patient records were reviewed for demographic information (gestational age (GA), sex, birth weight, PMA, weight at surgery, comorbidities, and preoperative oxygen/ventilator dependence). PMA was calculated as the sum of gestational age and chronological age. Respiratory comorbidities, specifically Bronchopulmonary Dysplasia (BPD) were stratified by severity according to the Jensen definitions [11]. Patients were classified as having grade 1 (mild), 2 (moderate), or 3 (severe) BPD based on their oxygen and respiratory support requirements preoperatively. Inguinal hernia repair is performed just prior to hospital discharge. Patients were selected for early surgical intervention if they presented with symptomatic herniation that necessitated repair before the criteria for discharge were met.

During hernia repair, all patients received general anesthesia. Standard anesthetic induction was achieved using Sevoflurane. Airway management consisted of endotracheal tube intubation. Regional anesthesia (caudal or ilioinguinal blocks) was not routinely performed. Hernia Perioperative data included operative time and hernia laterality. Length of postoperative hospitalization, respiratory complications, surgical complications, and recurrence rates were reviewed. Postoperative respiratory complications were defined as any event requiring an escalation of respiratory support within 6 h postoperatively. We stratified severity based on the maximum level of intervention required, categorized into three levels of clinical escalation: Grade I (mild): Requirement for supplemental oxygen above baseline to maintain saturation. Grade II (Moderate): Requirement for Non-Invasive Positive Pressure Ventilation (NIPPV), including CPAP or BiPAP. Grade III (Severe): Requirement for re-intubation or invasive mechanical ventilation (Advanced Airway). Surgical complications included wound infection, testicular atrophy, secondary high testes, hydrocele, bowel resection, accidental organ injury or recurrence. Recurrence was monitored for at least 12 months postoperatively.

Data analysis was conducted using IBM SPSS Statistics version 26.0. Categorical variables were assessed using the Chi-square and Fisher’s exact tests, while continuous variables were analyzed with one-way ANOVA. Univariate and multivariate logistic regression analyses were performed to identify independent risk factors for postoperative respiratory, recurrence and surgical complications. Statistical significance was set at *p* < 0.05, with a 95% confidence interval (CI) applied to the results.

## 3. Results

A total of 202 patients met the inclusion criteria and were categorized into two groups: early repair group: PMA < 38 weeks (*n* = 45) and delayed repair group: PMA ≥ 38 weeks (*n* = 157). Table 1 summarizes the demographic and clinical characteristics. The early group had a significantly lower mean gestational age than the late group (31.0 ± 2.7 weeks vs. 32.5 ± 3.6 weeks, *p* = 0.014). No significant differences were observed in the male-to-female ratio (23:22 vs. 94:63, *p* = 0.29) or birth weight (1624 ± 480 g vs. 1736 ± 664 g, *p* = 0.29). The early group had a lower body weight at surgery (2.5 ± 0.3 kg vs. 3.4 ± 0.9 kg, *p* < 0.001) and a higher prevalence of comorbidities (91.1% vs. 44.6%, *p* < 0.001). Respiratory comorbidities were significantly more prevalent in the Early Repair group compared to the Late Repair group (88.9% vs. 42.4%, *p* < 0.001). Specifically, the overall incidence of Bronchopulmonary Dysplasia (BPD) was higher in the Early group (40%) compared to the Late group (15.2%). Preoperative oxygen or ventilator dependence was similar between the two groups (24.4% vs. 17.8%, *p* = 0.32).

Table 2 presents perioperative findings and outcomes. Operative time was comparable between the groups (54.6 ± 20.1 min vs. 55.2 ± 23.1 min, *p* = 0.87). Bilateral hernias were prevalent in both groups (88.9% vs. 79%, *p* = 0.13). Postoperative respiratory complications were more common in the early group (20% vs. 7.6%, *p* = 0.02). This difference is driven primarily by Mild (Grade I) events (13.3% vs 3.8%, *p* = 0.03). Neither Moderate (Grade II) events (*p* = 1) nor Severe (Grade III) events (*p* = 0.19) showed a statistically significant difference between groups. Recurrence rates (2.2% vs. 1.3%, *p* = 0.64), surgical complication rates (4.4% vs. 6.4%, *p* = 0.63), postoperative ICU admissions (4.4% vs. 5.1%, *p* = 0.86), and hospitalization durations (3.2 ± 2.1 days vs. 3.1 ± 3.6 days, *p* = 0.83) were not significantly different.

The outcomes of the univariate and multivariate logistic regression analyses are detailed in Table 3 and Table 4. The univariate analysis identified PMA < 38 weeks, body weight < 2.8 kg at surgery, and preoperative oxygen dependence as significant risk factors for respiratory complications (*p* = 0.03, *p* = 0.007, and *p* < 0.001, respectively). Similarly, male sex, extremely low birth weight, and preoperative oxygen dependence were associated with surgical complications (*p* = 0.04, *p* = 0.02, *p* = 0.002). The multivariate analysis refined these findings: body weight < 2.8 kg and preoperative oxygen dependence remained the independent predictive factors for postoperative respiratory insufficiency (*p* = 0.04, *p* < 0.001). Furthermore, preoperative oxygen dependence was confirmed as a significant independent predictor for surgical complications (*p* = 0.04).

When comparing incarceration rates between the two groups, no statistically significant difference was observed (0% vs. 1.9%, *p* = 1). Three cases underwent emergent surgery due to irreducible bowel incarceration, all of which occurred at a postmenstrual age (PMA) of 40 weeks or above.

## 4. Discussion

The incidence of inguinal hernia (IH) is notably higher in premature infants, particularly in those with lower gestational ages (GA) and birth weight [1,2]. Surgical repairs of inguinal hernias are critical to prevent complications such as bowel incarceration; however, the timing of such repairs remains contentious due to the delicate balance between the risks of anesthesia and surgery versus the risk of incarceration. The lack of consensus regarding the optimal timing for elective inguinal hernia repair is evident from a survey conducted by the American Academy of Pediatrics [12], which reported that 63–70% of pediatric surgeons prefer to perform IHR before discharge from the neonatal intensive care unit (NICU), whereas others advocate for delaying surgery until the infant has achieved a more advanced postmenstrual age (PMA) and higher body weight. This variation highlights the complexity of the decision-making process and the need for evidence-based guidelines to optimize outcomes. Our surgical timing aligns with the ‘early repair’ strategy described in current literature, defined as operating prior to NICU discharge [13]. However, the clinical context of our cohort differs significantly due to our institution’s criteria for discharge readiness. In our practice, premature infants are weaned from the infant warmer at 2.2 kg and are considered ready for discharge upon reaching 2.5 kg. This threshold is lower than that of other institutions, where discharge or ‘pre-discharge’ surgery often occurs at higher weight milestones. This distinction underscores the necessity of this review: to validate the safety of inguinal hernia repair within this specific ‘early discharge, lower weight’ window.

One of the most significant concerns in performing early hernia repair is the increased risk of postoperative respiratory complications, particularly apnea. Premature infants are inherently at risk for apnea due to their neurological immaturity and underdeveloped respiratory systems, a phenomenon supported by the literature [14]. Rates of postoperative apnea in preterm infants may reach as high as 49%, with younger PMA strongly associated with a higher risk [15,16]. A recent large-scale randomized clinical trial reported that early hernia repair was associated with a significantly higher rate of serious adverse events compared to late repair (28% vs 18%). The authors suggested that delaying repair until after NICU discharge, specifically when infants are older than 55 weeks postmenstrual age, reduces the likelihood of these adverse events [13]. Our study corroborates these findings, demonstrating that infants who underwent inguinal hernia repair at a PMA of less than 38 weeks experienced significantly more respiratory complications compared to those in the delayed group. However, stratification by severity reveals that this statistical difference was driven primarily by Grade I (mild) events (13.3% vs 3.8%, *p* = 0.03). Importantly, we found no statistically significant difference in the incidence of moderate (Grade II) or severe (Grade III) complications between the two groups. This statistical distinction aligns with the clinical course observed, in our cohort, where respiratory insufficiency typically manifested as transient desaturation or brief apnea. Nearly 90% of these complications resolved within 48 h. and were managed with gentle stimulation, the temporary adjustment to a higher FiO_2_ setting or positive pressure ventilation support. Furthermore, a closer analysis of the Grade III (severe) events provides reassuring context: none of these patients required re-intubation due to respiratory failure after returning to the NICU. Instead, these cases comprised infants who were not extubated immediately in the operating room, for the purpose of precautionary observation and were successfully extubated once clinically stable. Only one patient in each group needed airway management that lasted for more than 48 h before returning to their clinical baseline respiratory parameters (including same mode of O_2_ support, same FiO_2_, PEEP, and same air flow rate). This also suggested that in our cohort, while early repair poses a heightened risk, the complications are often transient and manageable in a controlled hospital environment.

Risk factors for respiratory complications have been widely studied, with GA < 34 weeks and body weight < 2500 g consistently identified as significant predictors [16]. A multicenter retrospective review by Cho YJ et. al. found that early repair of inguinal hernia prior to NICU discharge significantly increases the risk of postoperative respiratory insufficiency [17]. Some articles suggested that infants with a PMA less than 44–46 weeks should be admitted for monitoring at least 12 h post-operatively. Between 46 and 60 weeks, the perioperative course must be individualized depending on PMA and comorbidity [18,19,20]. In our cohort, univariate analysis identified PMA < 38 weeks as a risk factor. However, multivariate analysis revealed that body weight < 2.8 kg and preoperatively oxygen dependence were the primary independent factors of respiratory complications. This statistical finding likely reflects the physiological link between somatic growth and respiratory maturation. Notably, we observed a significantly higher prevalence of mild BPD in the Early group (20%) compared to the Delayed group (3.2%), whereas rates of severe BPD remained relatively static or proportionally higher in the Delayed group. This suggests that infants in the Early group are often in a transitional phase of respiratory development. By delaying repair, these mild, reversible respiratory deficits have time to resolve, thereby reducing the risk of postoperative insufficiency. Conversely, the respiratory morbidity seen in the Delayed group is likely driven by chronic, severe lung disease that does not resolve with simple maturation. Furthermore, it is worth noting that while the Early group carried a significantly higher burden of non-respiratory comorbidities (including gastrointestinal, metabolic, and infectious histories), these factors did not emerge as independent predictors of respiratory complications in our multivariate analysis. This suggests that postoperative respiratory insufficiency is not merely a consequence of the ‘sicker’ overall profile of these infants, but is specifically driven by pulmonary immaturity and insufficient somatic growth. These findings align with existing evidence, reinforcing the importance of carefully considering these parameters when planning the timing of surgery.

Surgical outcomes, including complication and recurrence rates, are another critical consideration to the timing of inguinal hernia repair. Premature infants present unique surgical challenges due to the fragility of their tissues, the thinness of the hernia sac, and the potential for damage to the spermatic cord structures [21]. Nagraj et al. studied the incidence of complications after primary inguinal herniotomy in infants weighing ≤ 5 kg. It showed that the incidence of recurrence, testicular atrophy, and high testes requiring subsequent orchidopexy was 2.3%, 2.7%, and 2.7%, respectively [22]. For the overall rates of surgical complications in our study, despite the numbers being slightly higher compared to the previous study, the early group did not show an increased rate of surgical complications. Hernia recurrence in our study was comparable between the early and delayed repair groups, 2.2% vs 1.3% *p* = 0.64. This is consistent with previous studies, which report recurrence rates of 0.8–2.4% and suggest that the timing of surgery does not significantly affect recurrence rates. Furthermore, the low incidence of complications such as testicular atrophy, wound infection, and bowel injury in our cohort underscores the technical feasibility and safety of inguinal hernia repair in both early and delayed contexts, provided the procedure is performed by experienced pediatric surgeons.

In our cohort, surgery after 38 weeks PMA showed a trend towards higher risk of incarceration. Although statistical significance was not reached due to the low event rate, the clinical occurrence of incarceration exclusively in the delayed group highlights the possible risk of postponing repair. Among all the preterm infants, there were three patients in the latter group who presented in the emergency room with bowel incarceration (1.9%) prior to the scheduled elective operation. The postmenstrual age was 40, 40 and 41 respectively. Lautz et al. report that among premature infants, delaying hernia repair until after 40 weeks postmenstrual age is associated with higher incarceration rates, 21% compared with 11% at <36 weeks and 9% at 36–39 weeks [7]. In a separate study, de Goede et al. documented that very low birth weight infants exhibit an incarceration rate exceeding 50% when repair occurs within the first three months after birth. Emergent operations for incarcerated hernia bear a significantly higher recurrence and complication rates [23]. Since infants at the highest risk for postoperative respiratory events are typically already inpatients, the NICU environment provides an optimal window for safe surgical management. Performing early repair (PMA < 40 weeks) allows clinicians to address transient respiratory complications with established respiratory support protocols, thereby mitigating the significant risk of incarceration associated with delaying repair.

This retrospective review has several limitations. There was an underlying selection bias where only those who were diagnosed and referred by the neonatologists were included. Secondly, as a single-center study, the sample size was relatively small. This limited our statistical power, potentially leading to Type II errors where smaller differences between groups could not be detected. Thirdly, the limited sample size precluded the use of multivariate analysis to control for confounding factors. Specifically, while we identified respiratory conditions (such as high-grade respiratory distress syndrome, bronchopulmonary dysplasia, and recurrent pneumonia) as potential factors, we could not statistically isolate their independent role in postoperative respiratory insufficiency. Future prospective studies or multicenter studies with larger cohorts are necessary to validate these findings and allow for more robust statistical adjustment of these comorbidities.

## 5. Conclusions

In our cohort, early inguinal hernia repair in premature infants with a PMA of < 38 weeks is associated with a higher risk of postoperative respiratory complications. However, these complications are often mild and resolve within a short period of time. Preoperative oxygen dependence and body weight < 2.8 kg at the time of surgery are the main contributing risk factors. Furthermore, male sex, extremely low birth weight, and preoperative oxygen dependence showed a trend toward predicting surgical complications. The recurrence rate observed in this study was relatively low. Although not statistically significant, surgery after 40 weeks PMA shows a tendency towards higher risk of incarceration. We recommend that these clinical factors be carefully considered when selecting candidates for early repair to minimize the risk of postoperative complications.

## Figures and Tables

**Table 1:** Patient demographic characteristics.

	PMA ^2^ < 38 (*n* = 45)	PMA ≥ 38 (*n* = 157)	*p*
Gestational age (week)	31.0 ± 2.7	32.5 ± 3.6	0.014
Sex (M:F)	23:22	94:63	0.29
Birth weight (gram)	1624 ± 480	1736 ± 664	0.29
Postmenstrual age (week)	36.0 ± 0.9	40.9 ± 2.7	<0.001
Body weight (kilogram)	2.5 ± 0.3	3.4 ± 0.9	<0.001
Comorbidities	41 (91.1%)	70 (44.6%)	<0.001
Respiratory diseases	40 (88.9%)	67 (42.7%)	<0.001
Bronchopulmonary Dysplasia	18 (40.0%)	24 (15.2%)	<0.001
Mild	9 (20.0%)	5 (3.2%)	
Moderate	8 (17.8%)	11 (7.0%)	
Severe	1 (2.2%)	8 (5.1%)	
Cardiovascular diseases	12 (26.7%)	28 (17.8%)	0.19
Gastrointestinal diseases	23 (51.1%)	33 (21%)	<0.001
Urogenital diseases	2 (4.4%)	10 (6.4%)	0.63
Neurological diseases	9 (20%)	17 (10.8%)	0.11
Metabolic diseases	28 (62.2%)	38 (24.2%)	<0.001
Infections	20 (44.4%)	33 (21%)	<0.01
Genetic syndromes	0	2 (1.3%)	>0.999
Preoperative oxygen dependence/ventilator support	11 (24.4%)	28 (17.8%)	0.32
O_2_ cannula/hood	10 (22.2%)	19 (12.1%)	0.09
NIPPV ^1^	1 (2.2%)	6 (3.8%)	0.61
Mechanical ventilation	0	3 (1.9%)	>0.999

^1^ Non-invasive positive pressure ventilation. ^2^ Postmenstrual age.

**Table 2:** Perioperative findings and outcomes.

	PMA ^1^ < 38 (*n* = 45)	PMA ≥ 38 (*n* = 157)	*p*
Operation time (min)	54.6 ± 20.1	55.2 ± 23.1	0.87
Bilateral hernia	40 (88.9%)	124 (79%)	0.13
Unilateral hernia			
Right	5 (11.1%)	18 (22.5%)	0.95
Left	0	15 (9.6%)	0.03
Length of postoperative hospital stay (days)	3.2 ± 2.1	3.1 ± 3.6	0.83
Recurrence	1 (2.2%)	2 (1.3%)	0.64
Surgical complication	2 (4.4%)	10 (6.4%)	0.63
Wound infection	0	1 (0.6%)	>0.999
Testicular atrophy	0	0	>0.999
Secondary high testis	0	5 (3.2%)	0.59
Hydrocele	0	7 (4.5%)	0.35
Bowel resection	0	0	>0.999
Accidental injury of organs	1 (2.2%)	0	0.22
Postoperative respiratory insufficiency ^2^	9 (20%)	12 (7.6%)	0.02
Grade I (Mild)	6 (13.3%)	6 (3.8%)	0.03
Grade II (Moderate)	0	2 (1.3%)	1
Grade III (Severe)	3 (6.7%)	4 (2.5%)	0.19
Postoperative ICU admission	2 (4.4%)	8 (5.1%)	0.86

^1^ Postmenstrual Age. ^2^ Severity stratification of postoperative respiratory insufficiency: Grade I (mild): Requirement for supplemental oxygen above baseline to maintain saturation. Grade II (Moderate): Requirement for Non-Invasive Positive Pressure Ventilation (NIPPV), including CPAP or BiPAP. Grade III (Severe): Requirement for re-intubation or invasive mechanical ventilation (Advanced Airway).

**Table 3:** Factors associated with postoperative respiratory Insufficiency.

	Univariate Analysis		Multivariate Analysis	
	OR (95%Cl)	*p*	aOR ^2^ (95%Cl)	*p*
Sex, Male	2.08 (0.78–5.58)	0.14		
Postmenstrual age < 38 weeks	2.79 (1.10–6.98)	0.03	1.97 (0.60–6.44)	0.26
Birth weight < 1000 g	2.13 (0.76–5.91)	0.15		
Body weight at the time of surgery < 2.8 kg	3.70 (1.44–9.54)	0.007	3.36 (1.05–10.75)	0.04
Preoperative oxygen dependence/ventilator support	24.42 (8.19–72.79)	<0.001	27.04 (8.47–86.35)	<0.001
O_2_ cannula/hood	19.31 (6.03–61.86)	<0.001		
NIPPV ^1^/Mechanical ventilation	47.40 (10.09–222.66)	<0.001		

^1^ Non-invasive positive pressure ventilation, ^2^ Adjusted odd ratio: multivariate analysis adjusted for all variables with *p* < 0.05 in the univariate analysis (sex, birth weight, preoperative oxygen support, etc.).

**Table 4:** Factors associated with surgical complications.

	Univariate Analysis		Multivariate Analysis	
	OR (95%Cl)	*p*	aOR ^2^ (95%Cl)	*p*
Sex, Male	8.72 (1.10–68.88)	0.04	7.20 (0.89–58.39)	0.06
Postmenstrual age < 38 weeks	0.68 (0.14–3.24)	0.63		
Birth weight < 1000 g	4.13 (1.23–13.94)	0.02	1.94 (0.45–8.35)	0.37
Body weight at the time of surgery < 2.8 kg	1.07 (0.33–3.50)	0.91		
Preoperative oxygen dependence/ventilator support	6.91 (2.06–23.16)	0.002	4.30 (1.05–17.68)	0.04
O_2_ cannula/hood	5.06 (1.27–20.11)	0.02		
NIPPV ^1^/Mechanical ventilation	13.54 (2.68–68.38)	0.002		

^1^ Non-invasive positive pressure ventilation, ^2^ Adjusted odd ratio: multivariate analysis adjusted for all variables with *p* < 0.05 in the univariate analysis (sex, birth weight, preoperative oxygen support, etc.).

## Data Availability

The datasets generated and analyzed during the current study are available from the corresponding author upon reasonable request.
